# Relationship Between Prolonged Second Stage of Labor and Short-Term Neonatal Morbidity: A Systematic Review and Meta-Analysis

**DOI:** 10.3390/ijerph17217762

**Published:** 2020-10-23

**Authors:** Nuria Infante-Torres, Milagros Molina-Alarcón, Angel Arias-Arias, Julián Rodríguez-Almagro, Antonio Hernández-Martínez

**Affiliations:** 1 Mancha Centro Hospital, Av. Constitución, 3, Alcázar de San Juan, 13600 Ciudad Real, Spain; nuria.i.t@hotmail.com (N.I.-T.); angel_arias_arias81@hotmail.com (A.A.-A.); 2 Department of Nursing, Physiotherapy and Occupational Therapy, Faculty of Nursing, University of Castilla-La Mancha, Av. de España, s/n, 02001 Albacete, Spain; milagros.molina@uclm.es; 3 Department of Nursing, Physiotherapy and Occupational Therapy, Faculty of Nursing, University of Castilla-La Mancha, Camilo José Cela, 14, 13071 Ciudad Real, Spain; antonio.hmartinez@uclm.es

**Keywords:** Apgar score, meta-analysis, Neonatal Intensive Care Unit, neonatal morbidity, newborn care, labor stage, second, systematic review

## Abstract

To evaluate the association between prolonged second stage of labor and the risk of adverse neonatal outcomes with a systematic review and meta-analysis. PubMed, Scopus and EMBASE were searched using the search strategy “Labor Stage, Second” AND (length OR duration OR prolonged OR abnormal OR excessive). Observational studies that examine the relationship between prolonged second stage of labor and neonatal outcomes were selected. Prolonged second stage of labor was defined as 4 h or more in nulliparous women and 3 h or more in multiparous women. The main neonatal outcomes were 5 min Apgar score <7, admission to the Neonatal Intensive Care Unit, neonatal sepsis and neonatal death. Data collection and quality assessment were carried out independently by the three reviewers. Twelve studies were selected including 266,479 women. In nulliparous women, a second stage duration greater than 4 h increased the risk of 5 min Apgar score <7, admission to the Neonatal Intensive Care Unit and neonatal sepsis and intubation. In multiparous women, a second stage of labor greater than 3 h was related to 5 min Apgar score <7, admission to the Neonatal Intensive Care Unit, meconium staining and composite neonatal morbidity. Prolonged second stage of labor increased the risk of 5 min Apgar score <7 and admission to the Neonatal Intensive Care Unit in nulliparous and multiparous women, without increasing the risk of neonatal death. This review demonstrates that prolonged second stage of labor increases the risk of neonatal complications in nulliparous and multiparous women.

## 1. Introduction

The second stage of labor is the period of time between full cervical dilatation and birth of the baby, during which the woman has an involuntary urge to bear down, as a result of expulsive uterine contractions [1].

The description of the onset of the second stage of labor in clinical practice is often not precisely known. If complete dilatation is found on vaginal examination, it remains uncertain how long this cervical status has been present [2].

Multiple observational studies [2,3,4] have observed an increase in maternal complications associated with a prolonged second stage of labor, such as operative vaginal delivery, third-/fourth-degree perineal lacerations, caesarean delivery, urinary retention, postpartum hemorrhage and chorioamnionitis, as well as an increase in neonatal complications like seizures, hypoxic-ischemic encephalopathy, sepsis and increased mortality. However, the criteria these studies used to define the second stage of labor are heterogenous.

Thus, diagnosis and management of prolonged second stage of labor and its complications are difficult and often pose a dilemma to the obstetrician regarding timing and type of intervention [5]. Additionally, evidence on the duration of the second stage of labor is of very low certainty [1] and it is unclear whether there is a point of time from which the risk of perinatal complications increases and at which health professionals should intervene to prevent adverse events [3,6].

Nevertheless, there are professionals involved in childbirth care that try to reduce the duration of the second stage by obstetric interventionism in order to avoid neonatal complications. Paradoxically, these interventions, such as immediate pushing (initiated as soon as complete dilation is identified) [7], instrumental birth [8] or fundal pressure [9], may themselves increase the risk of neonatal morbidity.

In the past, a prolonged second stage of labor had been defined as a period of time that lasted beyond 2 h with epidural analgesia or 1 h without epidural analgesia for multiparous women. For nulliparous women, a prolonged second stage is defined as a period of time that lasted beyond 3 h with epidural analgesia or 2 h without epidural analgesia [10]. Recently, though, the American College of Obstetricians and Gynecologists (ACOG) [11] and the National Institute for Health and Care Excellence (NICE) [12] have allowed longer durations in specific cases. In spite of this, the correct management of the second stage of labor should be individualized according to birth progress, fetal malposition or the use of epidural analgesia [11,12]. For example, the Eunice Kennedy Shriver National Institute of Child Health and Human Development document suggested allowing one additional hour for the use of epidural analgesia. Thus, at least 3 h in multiparous women and 4 h in nulliparous women would be considered to diagnose a prolonged second stage of labor [11].

Thus, our objective was to evaluate the evidence on the association between prolonged second stage of labor (defined as 4 h in nulliparous women and 3 h in multiparous women) and the risk of adverse neonatal outcomes.

## 2. Materials and Methods

This systematic review with a meta-analysis was done according to PRISMA (Preferred Reporting Items for Systematic Review and Meta-Analyses) [13,14].

### 2.1. Data Sources and Searches

The adopted search strategy was: “Labor Stage, Second” (Mesh) AND (length OR duration OR prolonged OR abnormal OR excessive). Studies were identified in three main databases: PubMed [15], Scopus [16] and EMBASE [17], from 1 January 1990 to 1 November 2019. As well as published studies, we included non-published studies which had been included in the conference proceedings of the main scientific associations and indexed in the databases consulted. All languages were included. The search results for each database are provided in detail in Table A1.

All members of the research team had prior training in the methodology of systematic reviews, literature reviews and critical reading. AAA and AHM are also experts in meta-analysis.

Studies were included according to four criteria: (I) duration of second stage of labor greater than 4 h in nulliparous women; (II) duration of second stage of labor greater than 3 h in multiparous women; (III) studies reporting neonatal outcomes in relation to duration of second stage of labor; (IV) studies that stratified results by parity. Reference lists from the selected studies were also examined to locate further studies not identified using the search strategy. Two authors (NIT and AAA) independently performed the literature search and excluded any articles that did not meet the established inclusion criteria. A third author (MMA) was consulted to resolve any disagreements or uncertainty regarding inclusion.

### 2.2. Main Outcomes

The primary outcomes were 5 min Apgar score < 7, admission to the Neonatal Intensive Care Unit, neonatal sepsis and neonatal death. All neonatal outcomes examined by the available studies were included in this review. The definitions of some of the variables included in our study are shown in Table 1.

### 2.3. Data Extraction and Quality Assessment

Data collection and quality assessment were carried out independently by the three reviewers (NIT, AHM and JRA). We tried to contact the authors of several studies to provide us with data that did not appear in their manuscripts.

We used the Joanna Briggs Institute Critical Appraisal tools for use in JBI Systematic Reviews to assess the risk of bias in each included study [24]. Eleven domains were assessed to appraise the methodological quality of a study and to determine the extent to which a study had addressed the possibility of bias in its design, conduct and analysis.

### 2.4. Data Synthesis

For the categorical results, the odds ratio (OR) was used along with its 95% confidence intervals (95% CI). To calculate the OR, either the Mantel–Haenszel fixed-effects or Der Simonian–Laird random-effects models were used, depending on whether there was heterogeneity between the studies. Heterogeneity was assessed using the I^2^ and the statistical Cochran’s Q tests. I^2^ values of < 25%, 25–50 and >50% normally correspond to small, medium and large heterogeneity, respectively [14,25,26]. Publication bias was also evaluated using the Egger asymmetry test and funnel plots [14,27]. Statistical significance was defined at the ≤0.05 level.

All calculations were done with the StatsDirect statistical software, version 2.7.9. (Stats Direct Ltd., Cheshire, England) [14].

## 3. Results

### 3.1. Study Selection

**Figure 1:**
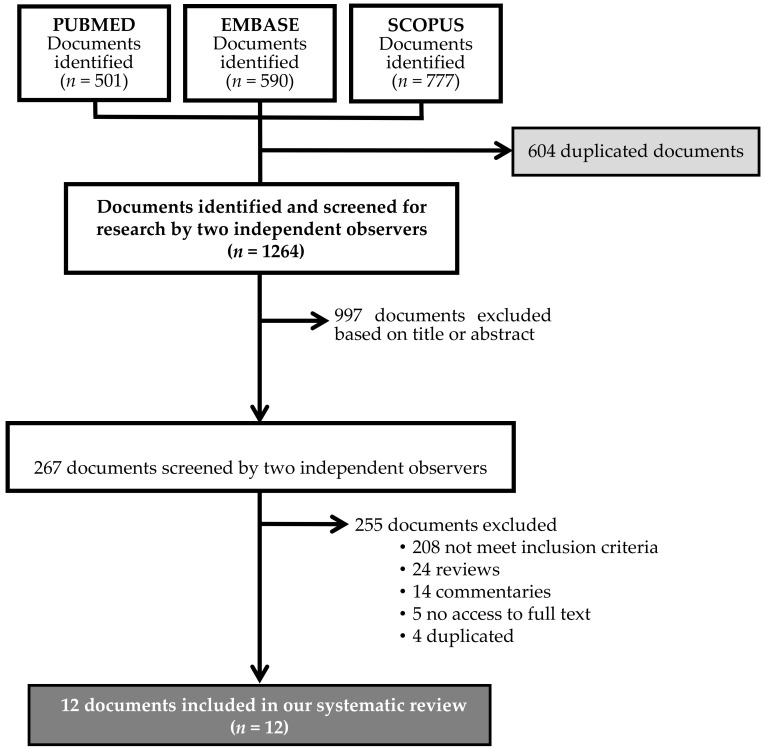
PRISMA flow diagram of the literature reviewing process.

A total of 1868 studies were selected from the literature search. After removing any duplicated articles, 267 were selected by title and abstract. After applying the inclusion/exclusion criteria, twelve articles were selected for the qualitative and quantitative analyses (meta-analysis) (Figure 1).

### 3.2. Study Characteristics

The description of the studies included in this systematic review are shown in Table 2. The sample included 268,624 women. The selected studies were conducted in Canada [4,18,28], United States [19,20,21,29,30], China [31], Sweden [22,32] and Spain [23]. The sample size of these studies ranged from 307 [31] to 121,490 [4]. All studies were restricted to singleton infants with cephalic presentation. Eight of these articles studied nulliparous women [18,20,21,22,28,30,31,32], two studied multiparous women [19,23] and two studied both (nulliparous and multiparous women) [4,29]

### 3.3. Study and Data Quality

The included studies had a low risk of bias, except for three studies that did not identify confounding factors [18,28,31] and four studies that did not include strategies to deal with confounding factors [18,28,30,31] (Table A2).

With regard to the selection of subjects, all studies except one [31] specified inclusion and exclusion criteria, selecting all women (nulliparas and/or multiparas) with singleton cephalic presentation that reached second stage of labor within a specific period of time.

Seven of the studies included in the meta-analysis [4,18,20,21,23,28,32] correctly defined prolonged second stage of labor (in this case, second stage of labor longer than 4 h in nulliparas and longer than 3 h in multiparas). Conversely, only three of them [18,21,28] established the maneuver used once prolonged second stage of labor was diagnosed (instrumental birth, continuing maternal pushing, caesarean, etc.).

As for data and information collection, five studies [4,22,23,30,32] included missing or incomplete data as exclusion criteria, so they were not included in the analysis.

### 3.4. Main Outcomes and Meta-Analysis

#### 3.4.1. Nulliparous Women

##### 5 min Apgar score <7

**Figure 2:**
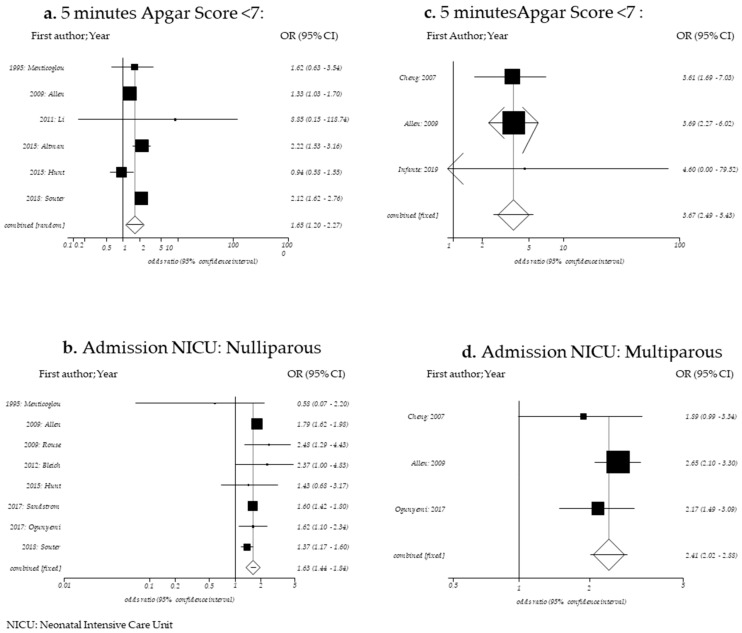
Forest plot for 5 min Apgar Score < 7 in nulliparous women (**a**), admission to NICU in nulliparous women (**b**), 5 min Apgar Score < 7 in multiparous women (**c**) and (**d**) admission to NICU in multiparous women.

To determine the relation between prolonged second stage of labor in nulliparous women (Table A3) and risk of low 5 min Apgar score (<7), six studies were included (*n* = 116,624) [4,18,28,30,31,32]. A significant increase in low 5 min Apgar score was observed when the second stage of labor lasted more than 4 h with respect to when the second stage of labor was ≤ 4 h. (OR = 1.65; 95% CI: 1.20–2.27). For this analysis, a random-effects model was used since heterogeneity was observed (Cochran’s Q *p*-value = 0.0041; I2 = 71.0) (Figure 2a; Table 3).

##### Admission to Neonatal Intensive Care Unit

To assess the risk of admission to the Neonatal Intensive Care Unit, eight studies were employed (n = 156,650) [4,18,20,21,22,28,29,30].

The risk significantly increased when the second stage of labor lasted more than 4 h with respect to when the second stage of labor was ≤ 4 h (OR, 1.63; 95%CI 1.44–1.84). For this analysis, a random-effects model was used since medium heterogeneity was observed (Cochran’s Q *p*-value = 0.057; I2 = 48.8) (Figure 2B; Table 3).

##### Neonatal Sepsis

By combining three studies (*n* = 82,053) [4,20,21], we found that the risk of neonatal sepsis increased when the duration of the second stage of labor was longer than 4 h with respect to when the second stage of labor was ≤ 4 h (OR, 1.57; 95% CI 1.07–2.29). For this analysis, a fixed-effects model was used since no heterogeneity was observed (Cochran’s Q *p*-value = 0.7962; I2 = 0.0) (Table 3).

##### Neonatal Death

Two studies (*n* = 28,032) [18,21] were employed to determine the relationship between prolonged second stage of labor and risk of neonatal death, and no differences were found (OR, 7.21; 95% CI 0.37–139.71) (Table 3).

##### Other Neonatal Outcomes

No significant associations were reported between prolonged second stage in nulliparous women and 1 min Apgar score < 1, 5 min Apgar score ≤ 3, umbilical artery pH < 7, acidosis, meconium-stained amniotic fluid, meconium aspiration, birth depression, minor or major trauma, birth trauma, shoulder dystocia, brachial plexus injury, Erb’s palsy, resuscitation at birth, heart compressions, hypoxic ischemic encephalopathy, hypothermia treatment or composite neonatal morbidity. When the results of two studies were combined [20,22], only an increased risk of neonatal intubation in women with a second stage of labor > 4 h was observed (OR, 2.19; 95% CI 1.23–3.90) (Table 3).

#### 3.4.2. Multiparous Women

##### 5 min Apgar Score < 7

To determine the relation between prolonged second stage of labor in multiparous women (Table A4) and risk of low 5 min Apgar score (< 7), three studies were included (*n* = 72,857) [4,19,23]. A significant increase in low 5 min Apgar score was observed when the second stage of labor lasted more than 3 h with respect to when the second stage of labor was ≤ 3 h (OR, 3.67; 95% CI 2.49–5.43). For this analysis, a fixed-effects model was used since no heterogeneity was observed (Cochran’s Q *p*-value = 0.987; I2 = 0.0) (Figure 2C; Table 3).

##### Admission to the Neonatal Intensive Care Unit

To assess the risk of admission to the Neonatal Intensive Care Unit, three studies were employed (*n* = 76,692) [4,19,29]. The risk significantly increased when the second stage of labor lasted more than 3 h with respect to when the second stage of labor was ≤ 3 h (OR, 2.41; 95% CI 2.02–2.88). For this analysis, a fixed-effects model was used since no heterogeneity was observed (Cochran’s Q *p*-value = 0.417; I2 = 0.0) (Figure 2D; Table 3).

##### Neonatal Sepsis

None of the studies that analyzed multiparous women considered this variable when assessing neonatal morbidity in relation to the duration of the second stage of childbirth (Table 3).

##### Neonatal Death

None of the studies that analyzed multiparous women considered this variable when assessing neonatal morbidity in relation to the duration of the second stage of childbirth (Table 3).

##### Other Neonatal Outcomes

No significant associations were reported between prolonged second stage in multiparous women and umbilical artery pH < 7.0, umbilical artery pH < 7.10, umbilical artery base excess ≥12, meconium aspiration, shoulder dystocia, prolonged neonatal stay, advanced neonatal resuscitation, birth depression, minor or major trauma or any perinatal morbidity. After combining two studies [19,29], only an increase in the risk of meconium staining was observed (OR, 1.29; 95%CI, 1.01–1.66), and an increase in composite neonatal morbidity (OR,1.97; 95% CI, 1.39–2.80) was observed after another two studies were combined [19,23] (Table 3).

#### 3.4.3. Publication Bias

We did not observe publication bias for the study in any of the variables studied (Table A3 and Table A4).

We can observe a summary of results obtained following meta-analysis of all variables studied in nulliparous and multiparous women in Table 3.

## 4. Discussion

### 4.1. Main Findings

Our meta-analysis results suggested that duration of second stage of labor of more than 4 h in nulliparous women increased the risk of low 5 min Apgar score < 7, admission to the Neonatal Intensive Care Unit, neonatal sepsis and neonatal intubation. In multiparous women, when the second stage of labor was longer than 3 h, the risk of 5 min Apgar score < 7, admission to Neonatal Intensive Care Unit, meconium staining and composite neonatal morbidity increased.

However, a prolonged second stage of labor did not increase the risk of any of the other variables studied, such as umbilical artery pH < 7, birth depression, neonatal death meconium aspiration or shoulder dystocia.

### 4.2. Comparison with Existing Literature

The literature has very limited data on neonatal outcomes of women with duration of second stage of labor of more than 4 h in nulliparas and of more than 3 h in multiparas. We were only able to locate 12 articles with these durations for this review.

An example of this is a recent systematic review by Gimovksy et al., which evaluated the maternal and fetal morbidities associated with prolonged second stage of labor in nulliparous women with epidurals, in which the authors defined prolonged second stage as greater than three hours [33]. Only two papers were included in this systematic review, and very discordant neonatal outcomes were analyzed, which did not allow the results to be combined in order to establish conclusions that would be useful for decision-making in clinical practice.

Another systematic review studied the influence of prolonged second stage of labor on the risk of adverse maternal and neonatal outcomes from 1980 until 2005 [34]. It did not report associations between prolonged second stage and adverse neonatal outcomes, but most of the studies analyzed in this review defined the prolongation of the second stage as more than 2 h, without differentiating according to parity. In addition, it did not conform to the new recommendations of allowing longer durations.

Only one randomized controlled trial [35] specifically addressed the effect of this change in obstetric practice on maternal and neonatal outcomes. In that trial, a policy of extending the second stage of labor for at least 1 h in nulliparous women with epidural anesthesia with respect to “usual labor” (3 h) decreased the incidence of caesarean birth by more than half compared with the common practice (19.5%, 8 of 41, vs. 43.2%, 16 of 37; RR, 0.45; 95% CI, 0.22–0.93). Maternal or neonatal morbidity were not statistically different between the groups. Unfortunately, the trial was underpowered to detect significant differences in the frequency of adverse maternal or neonatal outcomes between groups because the sample studied was very small (only 78 nulliparous women) (35).

However, Zipori et al. [36] recently published another study comparing maternal and neonatal outcomes over two distinct time periods. In period I, the duration of the second stage of labor was considered prolonged according to ACOG limits, and it was called a “classic labor curve” (10). The “new labor curve” of period II allowed nulliparous and multiparous women to continue the second stage of labor for an additional 1 h before diagnosing second-stage arrest. Primary caesarean deliveries decreased with the new policy of labor management, with a small rise in instrumental deliveries, but it also increased other immediate maternal and neonatal complications, such as higher rate of lower umbilical artery cord pH.

### 4.3. Strengths and Limitations

One of the strengths of this study is that it is the first systematic review to define prolonged second stage of labor according the most recent recommendations (11), that is, 4 h for nulliparous women and 3 h for multiparous women. Most of the studies had large sample sizes with sufficient numbers of participants in each group to lend power to the findings, and the majority of them used methods to control for potential confounding factors.

Among the limitations of our systematic review is that neonatal outcome measures were discordant in the included studies, meaning it was difficult to combine data to summarize important clinical findings, and that the definition of two variables (admission to NICU and composite neonatal morbidity) differed among included studies. None of the studies considered the pushing duration or pushing techniques employed (delayed pushing or immediate pushing). Finally, since they were observational studies, there is a risk of confounding bias even though many of the studies included techniques to control confounding. 

## 5. Conclusions

In nulliparous women, a prolonged second stage of labor is not related with an increased risk of neonatal death. However, it is related with an increased risk of 5 min Apgar score < 7, admission to the Neonatal Intensive Care Unit, neonatal sepsis or intubation. In multiparous women, a prolonged second stage of labor is related with an increased risk of 5 min Apgar score < 7, admission to the Neonatal Intensive Care Unit, meconium staining and composite neonatal morbidity.

These potential risks associated with a prolonged second stage of labor in both nulliparous and multiparous women should serve as an incentive for professionals involved in childbirth care to increase supervision of mothers who exceed these durations.

More studies are needed, especially clinical studies, to guarantee the safety of newborns when the second stage of labor exceeds 4 h in nulliparous women and 3 h in multiparous women.

## Figures and Tables

**Table 1:** Definition of variables.

Definitions	Authors						
	1995; Menticoglou [18]	2007; Cheng [19]	2009; Allen [4]	2009; Rouse [20]	2012; Bleich [21]	2017; Sandström [22]	2019; Infante [23]
Acidosis	NR	NR	NR	NR	NR	A pH value <7.05 and base excess <−12 in the umbilical artery.	NR
Birth depression	NR	NR	Delay in initiating and maintaining respirations after birth requiring resuscitation by mask or endotracheal tube for at least 3 min, a 5 min Apgar score of 3 or less, or neonatal seizures due to hypoxic-ischemic encephalopathy.	NR	NR	NR	NR
Intubation	NR	NR	NR	Intubation in delivery room.	NR	NR	NR
Heart compressions	NR	NR	NR	NR	NR	Resuscitation in delivery room with heart compressions and/or intubation.	NR
Advanced neonatal resuscitation	NR	NR	NR	NR	NR	NR	Type III: Oxygen therapy with positive intermittent pressure. Type IV: Endotracheal intubation, Type V: Cardiac massage and/or using drugs
Admission to Neonatal Intensive Care Unit	Need for admission to the Neonatal Intensive Care Unit for any reason at all or with a 5 min Apgar score < 7 or arterial cord pH < 7.20.	NR	Neonatal intensive care unit admission with duration of stay longer than 24 h.	Admission to a neonatal intensive care unit for >48 h.	NR	NR	NR
Prolonged neonatal stay	NR	Neonatal stay >2 d for vaginal delivery and >4 d for caesarean delivery.	NR	NR	NR	NR	NR
Neonatal seizures	NR	NR	NR	NR	Seizures in the first 24 h of life.	NR	NR
Sepsis	NR	NR	Positive blood culture, septicemia or systematic infection.	NR	Positive blood culture.	NR	NR
Minor trauma	NR	NR	One or more of the following neonatal traumas: linear skull fracture, other fractures (clavicle, ribs, numerus, or femur), facial palsy, or cephalohematoma.	NR	NR	NR	NR
Major trauma	NR	NR	One or more of the following neonatal traumas: depressed skull fracture, intracranial hemorrhage, or brachial plexus palsy.	NR	NR	NR	NR
Composite neonatal morbidity	NR	Composite variable for 5 min Apgar <7, UA pH <7.0, UA base excess ≥12, shoulder dystocia, NICU stay, and birth trauma (which includes brachial plexus injury, facial nerve palsy, clavicular fracture, skull fracture, head laceration, and cephalohematoma defined and diagnosed by the attending pediatrician).	Composite of any of the other neonatal outcomes.	Any of the following occurrences: a 5 min Apgar score <4, an umbilical artery pH <7.0, seizures, intubation, stillbirth, neonatal death, or admission to a NICU.	NR	NR	Composite of any of the other neonatal outcomes.
Neonatal death	Death during the second stage of labor or in the first 28 d of life	NR	NR	NR	NR	NR	NR

NR: not reported.

**Table 2:** Characteristics of the studies analyzed.

	YEAR OF PUBLICATION AUTOR	COUNTRY	STUDY DESIGN	POPULATION UNDER STUDY	DURATION OF SECOND STAGE OF LABOUR *n* (%)					DELIVERY MODE N (%)			USE OF EA N (%)	INCLUSION/EXCLUSION CRITERIA
					0–1 h	1–2 h	2–3 h	3–4 h	>4 h	Spontaneous Vaginal Delivery	Operative Vaginal Delivery	Caesarean Section		
**NULLIPAROUS**	**1995/** **Menticoglou** [18]	Canada	Cohort study	6041	2622 (43.4)	1805 (29.9)	927 (15.3)	379 (6.3)	308 (5.1)	4942 (81.8)	932 (15.5)	167 (2.7)	NR	January 1988 to December 1992. Singleton babies in cephalic presentation. BW ≥ 2500 g Fetal death diagnosed before labor and caesarean section before labor or during the first stage of labor were excluded.
	**2009/** **Rouse** [20]	United States	Secondary analysis of a clinical trial	4126	1901 (46.1)	1251 (30.3)	614 (14.9)	217 (5.2)	143 (3.5)	3054 (74.0)	765 (18.5)	307 (7.5)	3916 (95.0)	Nulliparous women with a singleton vertex fetus who labored spontaneously or were induced at ≥ 36 WG and who reached the second stage of labor. Exclusion criteria included maternal fever and serious medical conditions.
	**2011/** **Li** [31]	China	Case-control study	307	206 (67.1)		29 (9.4)	60 (19.5)	12 (4.0)	NR	NR	NR	NR	NR
	**2012/** **Bleich** [21]	United States	Cohort study	21,991	13,736 (62.5)	4933 (22.4)	1833 (8.3)	1062 (4.8)	427 (2.0)	19,326 (87.9)	1367 (6.2)	1298 (5.9)	13,676 (62.2)	Nulliparous women who reached the second stage of labor. Singleton live-born infants at ≥ 37 WG and cephalic presentation. Between January 2003 to December 2008. Fetal malformations, placenta previa and multiple gestation were excluded.
	**2015/** **Altman** [32]	Sweden	Cohort study	32,796	10,731 (32.7)	9491 (29.0)	5856 (17.8)	3898 (11.9)	2820 (8.6)	NR	6728 (20.5)	NR	19,417 (59.2)	First live singleton infant in cephalic presentation at ≥ 37 WG. From January 2008 to December 2012. Caesarean and induced deliveries and deliveries with incomplete data were excluded.
	**2015/** **Hunt** [28]	Canada	Cohort study	1515	NC	NC	NC	629 (41.5)	886 (58.5)	615 (40.6)	662 (43.7)	238 (15.7)	NR	Nulliparous women who delivered non-anomalous, term (≥ 36 WG), cephalic, live singleton neonatal weight ≥ 2500 g and who had a prolonged second stage of labor. Between January 1993 and April 2006.
	**2017/** **Sandström** [22]	Sweden	Cohort study	42,539	13,558 (31.9)	12,225 (28.7)	7710 (18.1)	5238 (12.3)	3808 (9.0)	NR	NR	NR	NR	Between January 2008 to December 2013. Nulliparous women with cephalic presentation at 37 WG or later. Elective caesarean deliveries, emergency caesareans during first stage of labor and deliveries with incomplete data were excluded (without labor partograph or notation on complete dilation of the cervix).
	**2018/** **Souter** [30]	United States	Cohort study in a poster session	20,029	16,682 (83.3)			3347 (16.7)		14,942 (74.6)	3015 (15.0)	2072 (10.4)	20,029 (100)	Singleton deliveries at 37 + 0 to 42 + 6 WG between January 2012 and December 2016
**MULTIPAROUS**	**2007/** **Cheng** [19]	United States	Cohort study	5158	4112 (79.7)	550 (10.7)	239 (4.6)	257 (5.0)		4480 (86.8)	414 (8.1)	263 (5.1)	2274 (44.1)	Between 1991 and 2001. All term and post-term, cephalic, live singleton births to multiparous women who had spontaneous onset of labor. Caesarean delivery before the completion of the first stage of labor, placenta previa, intrauterine fetal demise or known lethal congenital anomalies were excluded.
	**2019/** **Infante** [23]	Spain	Cohort study	2145	1589 (74.1)	327 (15.2)	165 (7.7)	64 (3.0)		2070 (96.5)	75 (3.5)	NR	1675 (78.1)	Women who had given birth vaginally, with cephalic presentation and singleton babies between 2013 and 2016. Births with < 35 WG and antepartum fetal death were excluded.
**NULLIPAROUS AND MULTIPAROUS**	**2009/** **Allen** [4]	Canada	Cohort study	55,936 nulliparous	38,790 (69.3)		7832 (14.0)	4406 (7.9)	4908 (8.8)	101,897 (83.8)	15,865 (13.1)	3734 (3.1)	61,077 (50.3)	Between 1988 and 2006. Liveborn singleton at or after 37 WG reaching full cervical dilatation. Deliveries that occurred before onset of labor, a major congenital anomaly, at least one previous caesarean delivery, severe pregnancy-related medical disorders or missing outcome data were excluded
				65,554 multiparous	59,227 (90.3)	4171 (6.3)	1188 (1.8)	968 (1.6)						
	**2017/** **Ogunyemi** [29]	United States	Poster session	10,487 *	NC	NC	NC	NC	NC	NR	NR	NR	NR	Singleton at term.

NC: not calculated, NR: not reported, EA: epidural analgesia, BW: birthweight, WG: weeks gestation, *: no data on nulliparous/multiparous.

**Table 3:** Summary of results obtained following meta-analysis of all variables studied in nulliparous women. Summary of results obtained following meta-analysis of all variables studied in multiparous women.

Variable	Number of Studies	Number of Subjects	Egger Bias (*p*-Value)	I^2^ 95% CI	Cochran’s Q (*p*-Value)	OR 95% CI
1 min Apgar Score <7	1	307	NC	NC	NC	NC
5 min Apgar score <7	6	116,624	0.7861	71.0 (2,1–85,6)	0.0041	1.65 (1.20–2.27)
5 min Apgar score <4	2	36,922	NC	NC	0.7026	2.27 (1.08–4.74)
5 min Apgar Score <3	1	21,991	NC	NC	NC	NC
Umbilical artery pH <7	2	29,117	0.8132	NC	0.8132	2.30 (0.94–5.69)
Umbilical artery pH <7.10	0	0	NR	NR	NR	NR
Umbilical artery base excess >−12	0	0	NR	NR	NR	NR
Acidosis	1	33,429	NC	NC	NC	NC
Birth depression	1	55,936	NC	NC	NC	NC
Resuscitation at delivery	2	42,020	NC	NC	<0.001	2.60 (0.81–8.63)
Intubation	2	46,665	NC	NC	0.681	2.19 (1.23–3.90)
Heart compressions	1	42,539	NC	NC	NC	NC
ANR	0	0	NR	NR	NR	NR
Meconium aspiration	1	42,539	NC	NC	NC	NC
Meconium-stained amniotic fluid	1	4487	NC	NC	NC	NC
Admission to Neonatal Intensive Care Unit	8	156,650	0.8326	48.8 (0.0–75.4)	0.0573	1.63 (1.44–1.84)
Prolonged neonatal stay	0	0	NR	NR	NR	NR
Neonatal seizures	3	70,571	NC	92.3 (78.6–95.8)	<0.001	4.67 (0.78–27.78)
Neonatal sepsis	3	82,053	NC	0.0 (0–72.9)	0.7962	1.57 (1.07–2.29)
Birth trauma	1	4064	NC	NC	NC	NC
Minor trauma	1	55,936	NC	NC	NC	NC
Major trauma	1	55,936	NC	NC	NC	NC
Shoulder dystocia	1	20,029	NC	NC	NC	NC
Brachial plexus injury	1	4126	NC	NC	NC	NC
Erb’s palsy	1	21,991	NC	NC	NC	NC
Hypoxic ischemic encephalopathy	1	42,539	NC	NC	NC	NC
Hypothermia treatment	1	42,539	NC	NC	NC	NC
Composite neonatal morbidity	1	4126	NR	NR	NR	NR
Any perinatal morbidity	0	0	NR	NR	NR	NR
Neonatal death	2	28,032	NC	NC	NC	7.21 (0.37–139.71)
**Variable**	**Number of Studies**	**Number of Subjects**	**Egger Bias** **(*p*-Value)**	**I^2^ 95% CI**	**Cochran’s Q** **(*p*-Value)**	**OR 95% CI**
1 min Apgar Score < 7	0	0	NR	NR	NR	NR
5 min Apgar score < 7	3	72,857	NC	0.0 (0.0–72.9)	0.987	3.67 (2.48–5.43)
5 min Apgar score < 4	0	0	NR	NR	NR	NR
5 min Apgar Score ≤ 3	0	0	NR	NR	NR	NR
Umbilical artery pH < 7	1	5158	NC	NC	NC	NC
Umbilical artery pH < 7.10	1	1912	NC	NC	NC	NC
Umbilical artery base excess > −12	1	5158	NC	NC	NC	NC
Acidosis	0	0	NR	NR	NR	NR
Birth depression	1	65,554	NC	NC	NC	NC
Resuscitation at delivery	0	0	NR	NR	NR	NR
Intubation	0	0	NR	NR	NR	NR
Heart compressions	0	0	NR	NR	NR	NR
ANR	1	2145	NC	NC	NC	NC
Meconium aspiration	0	0	NR	NR	NR	NR
Meconium-stained amniotic fluid	2	11,193	NC	NC	0.121	1.29 (1.01–1.66)
Admission to Neonatal Intensive Care Unit	3	76,692	NC	0.0 (0.0–72.9)	0.417	2.41 (2.02–2.88)
Prolonged neonatal stay	1	5158	NC	NC	NC	NC
Neonatal seizures	0	0	NR	NR	NR	NR
Neonatal sepsis	0	0	NR	NR	NR	NR
Birth trauma	0	0	NR	NR	NR	NR
Minor trauma	1	65,554	NC	NC	NC	NC
Major trauma	1	65,554	NC	NC	NC	NC
Shoulder dystocia	1	5158	NC	NC	NC	NC
Brachial plexus injury	0	0	NR	NR	NR	NR
Erb’s palsy	0	0	NR	NR	NR	NR
Hypoxic ischemic encephalopathy	0	0	NR	NR	NR	NR
Hypothermia treatment	0	0	NR	NR	NR	NR
Composite neonatal morbidity	2	7303	NC	NC	0.330	1.97 (1.39–2.80)
Any perinatal morbidity	1	65,554	NC	NC	NC	NC
Neonatal death	0	0	NR	NR	NR	NR

ANR: advanced neonatal resuscitation; NC: not calculated; NR: not reported.

## Appendix A

**Table A1:** Search strategies.

Search Strategies		
Search Strategy	Database	Hits
“Labor Stage, Second” (Mesh) AND (length OR duration OR prolonged OR abnormal OR excessive)	PubMed	501
	Scopus	590
	Embase	777
**Search Strategy “PICO”**		
Population	Nulliparous and Multiparous women	
Intervention	Second stage labor > 4 h in nulliparous Second stage labor > 3 h in multiparous	
Comparison	Second stage labor ≤ 4 h in nulliparous Second stage labor ≤ 3 h in multiparous	
Outcome	Neonatal morbidity: 5 min Apgar score < 7, admission to the Neonatal Intensive Care Unit, neonatal sepsis and neonatal death.	

**Table A2:** Checklist for Cohort Studies.

.	1995; Menticoglou	2007; Cheng	2009; Allen	2009; Rouse	2011; Li	2012; Bleich	2015; Altman	2015; Hunt	2017; Sandström	2017; Ogunyemi	2018; Souter	2019; Infante
1. Were the two groups similar and recruited from the same population?	Unclear *	No *	No *	Unclear *	Unclear *	Unclear *	Unclear *	Unclear *	Unclear *	Unclear *	Unclear *	Unclear *
2. Were the exposures measured similarly to assign people to both exposed and unexposed groups?	Yes	Yes	Yes	Yes	Yes	Yes	Yes	Yes	Yes	Yes	Yes	Yes
3. Was the exposure measured in a valid and reliable way?	Yes	Yes	Yes	Yes	No	Yes	Yes	Yes	Yes	Yes	Yes	Yes
4. Were confounding factors identified?	No	Yes	Yes	Yes	No	Yes	Yes	No	Yes	Yes	Yes	Yes
5. Were strategies to deal with confounding factors stated?	No	Yes	Yes	Yes	No	Yes	Yes	No	Yes	Yes	No	Yes
6. Were the groups/participants free of the outcome at the start of the study (or at the moment of exposure)?	Yes	Yes	Yes	Yes	Yes	Yes	Yes	Yes	Yes	Yes	Yes	Yes
7. Were the outcomes measured in a valid and reliable way?	Yes	Yes	Yes	Yes	Yes	Yes	Yes	Yes	Yes	Yes	Yes	Yes
8. Was the follow up time reported and sufficient to be long enough for outcomes to occur?	Yes	Yes	Yes	Yes	Yes	Yes	Yes	Yes	Yes	Yes	Yes	Yes
9. Was follow up complete, and if not, were the reasons to loss to follow up described and explored?	Yes	Yes	Yes	Yes	Yes	Yes	Yes	Yes	Yes	Yes	Yes	Yes
10. Were strategies to address incomplete follow up utilized?	NA	NA	Yes	NA	NA	NA	Yes	NA	Yes	NA	NA	Yes
11. Was appropriate statistical analysis used?	Unclear	Yes	Yes	Yes	Unclear	Yes	Yes	Unclear	Yes	Yes	Unclear	Yes

NA: not applicable, *: groups recruited from the same population.

**Table A3:** Neonatal morbidity outcomes in nulliparous women (≤4 h versus >4 h second-stage labor).

	1 min Apgar Score <7		5 min Apgar Score <7		5 min Apgar Score <4		5 min Apgar Score ≤3		Umbilical Artery pH <7		Acidosis		Birth Depression		Resuscitation at Delivery			Intubation		Heart Compressions	
Author	≤4 h	>4 h	≤4 h	>4 h	≤4 h	>4 h	≤4 h	>4 h	≤4 h	>4 h	≤4 h	>4 h	≤4 h	>4 h	≤4 h		>4 h	≤4 h	>4 h	≤4 h	>4 h
1995; Menticoglou	NR	NR	81/5733	7/308	NR	NR	NR	NR	NR	NR	NR	NR	NR	NR	NR		NR	NR	NR	NR	NR
2009; Allen	NR	NR	589/51,028	75/4908	NR	NR	NR	NR	NR	NR	NR	NR	862/51,028	138/4908	NR		NR	NR	NR	NR	NR
2009; Rouse	NR	NR	NR	NR	3/3983	0/143	NR	NR	15/3983	1/143	NR	NR	NR	NR	NR		NR	19/3983	1/143	NR	NR
2011; Li	17/295	8/12	3/295	1/12	NR	NR	NR	NR	NR	NR	NR	NR	NR	NR	NR		NR	NR	NR	NR	NR
2012; Bleich	NR	NR	NR	NR	NR	NR	17/21,564	2/427	83/21,564	4/427	NR	NR	NR	NR	138/21,564		13/427	NR	NR	NR	NR
2015; Altman	NR	NR	188/29,976	39/2820	32/29,976	8/2820	NR	NR	NR	NR	NR	NR	NR	NR	NR		NR	NR	NR	NR	NR
2015; Hunt	NR	NR	33/629	44/886	NR	NR	NR	NR	NR	NR	NR	NR	NR	NR	NR		NR	NR	NR	NR	NR
2017; Sandström	NR	NR	NR	NR	NR	NR	NR	NR	NR	NR	330/30,453	31/2976	NR	NR	NR		NR	58/38,731	13/3808	36/38,731	10/3808
2017; Ogunyemi	NR	NR	NR	NR	NR	NR	NR	NR	NR	NR	NR	NR	NR	NR	NR		NR	NR	NR	NR	NR
2018; Souter	NR	NR	200/16,682	84/3347	NR	NR	NR	NR	NR	NR	NR	NR	NR	NR	851/16,682		248/3347	NR	NR	NR	NR
Egger Bias (*p*-value)			0.7861		NC				0.8132						NC			NC			
I^2^ 95% CI			71.0 (2.1–85.6)		NC				NC						NC			NC			
Q Cochran (*p*-value)			0.0041		0.7026				0.8132						<0.001			0.681			
OR 95% CI			**1.65 (1,20–2.27) ***		**2.27 (1.08–4.74) ***				2.30 (0.94–5.69)						2.60 (0.81–8.63) *			**2.19 (1.23–3.90)**			
	**Meconium Aspiration**		**Meconium-Stained Amniotic Fluid**		**Admission to NICU**				**Neonatal Seizures**		**Sepsis**		**Birth Trauma**		**Minor Trauma**			**Major Trauma**		**Shoulder Dystocia**	
**Author**	**≤4 h**	**>4 h**	**≤4 h**	**>4 h**	**≤4 h**		**>4 h**		**≤4 h**	**>4 h**	**≤4 h**	**>4 h**	**≤4 h**	**>4 h**	**≤4 h**		**>4 h**	**≤4 h**	**>4 h**	**≤4 h**	**>4 h**
1995; Menticoglou	NR	NR	NR	NR	64/5733		2/308		5/5733	0/308	NR	NR	NR	NR	NR		NR	NR	NR	NR	NR
2009; Allen	NR	NR	NR	NR	3071/51,028		505/4908		NR	NR	195/51,028	30/4908	NR	NR	1311/51,028		162/4908	78/51,028	16/4908	NR	NR
2009; Rouse	NR	NR	NR	NR	167/3983		14/143		NR	NR	6/3983	0/143	NR	NR	NR		NR	NR	NR	NR	NR
2011; Li	NR	NR	NR	NR	NR		NR		NR	NR	NR	NR	NR	NR	NR		NR	NR	NR	NR	NR
2012; Bleich	NR	NR	NR	NR	172/21,564		8/427		41/21,564	13/427	39/21,564	0/427	NR	NR	NR		NR	NR	NR	NR	NR
2015; Altman	NR	NR	NR	NR	NR		NR		NR	NR	NR	NR	NR	NR	NR		NR	NR	NR	NR	NR
2015; Hunt	NR	NR	NR	NR	12/629		24/886		NR	NR	NR	NR	NR	NR	NR		NR	NR	NR	NR	NR
2017; Sandström	58/38,731	10/3808	NR	NR	2373/38,731		360/3808		78/38,731	17/3808	NR	NR	NR	NR	NR		NR	NR	NR	NR	NR
2017; Ogunyemi	NR	NR	309/4215	37/272	372/4201		37/272		NR	NR	NR	NR	33/3814	1/250	NR		NR	NR	NR	NR	NR
2018; Souter	NR	NR	NR	NR	817/16,682		221/3347		NR	NR	NR	NR	NR	NR	NR		NR	NR	NR	367/16,682	74/3347
Egger Bias (*p*-value)					0.8326				NC		NC										
I^2^ 95% CI					48.8 (0.0–75,4)				92.3 (78.6–95,8)		0.0 (0–72.9)										
Q Cochran (*p*-value)					0.0573				<0.001		0.7962										
OR 95% CI					**1.63 (1.53–1.74)**				4.67 (0,78–27.78) *		**1.57 (1.07–2.29)**										
	**Brachial Plexus Injury**		**Erb’s Palsy**		**Hypoxic Ischemic Encephalopathy**						**Hypothermia Treatment**				**Composite Neonatal Morbidity**				**Neonatal Death**		
**Author**	**≤4 h**	**>4 h**	**≤4 h**	**>4 h**	**≤4 h**				**>4 h**		**≤4 h**		**>4 h**		**≤4 h**	**>4 h**			**≤4 h**	**>4 h**	
1995; Menticoglou	NR	NR	NR	NR	NR				NR		NR		NR		NR	NR			0/5733	0/308	
2009; Allen	NR	NR	NR	NR	NR				NR		NR		NR		NR	NR			NR	NR	
2009; Rouse	10/3983	1/143	NR	NR	NR				NR		NR		NR		98/3983	6/143			NR	NR	
2011; Li	NR	NR	NR	NR	NR				NR		NR		NR		NR	NR			NR	NR	
2012; Bleich	NR	NR	82/21,564	2/427	NR				NR		NR		NR		NR	NR			3/21,564	0/427	
2015; Altman	NR	NR	NR	NR	NR				NR		NR		NR		NR	NR			NR	NR	
2015; Hunt	NR	NR	NR	NR	NR				NR		NR		NR		NR	NR			NR	NR	
2017; Sandström	NR	NR	NR	NR	75/38,731				22/3808		16/38,731		7/3808		NR	NR			NR	NR	
2017; Ogunyemi	NR	NR	NR	NR	NR				NR		NR		NR		NR	NR			NR	NR	
2018; Souter	NR	NR	NR	NR	NR				NR		NR		NR		NR	NR			NR	NR	
Egger Bias (*p*-value)																			NC		
I^2^ 95% CI																			NC		
Q Cochran (*p*-value)																			NC		
OR 95% CI																			7.21 (0.37–139.71) *		

NR: not reported; NR: not calculated; CI: confidence interval; * Random effects (DerSimonian–Laird); Bold: Significant results are highlighted.

**Table A4:** Neonatal Morbidity Outcomes in Multiparous Women (≤3 h versus >3 h second stage of labour).

	5 min Apgar Score <7		Umbilical Artery pH <7.0		Umbilical Artery pH <7.10		Umbilical Artery Base Excess>−12		Birth Depression		Advanced Neonatal Resuscitation		Meconium Amniotic Fluid or Meconium Staining		NICU Admission		Prolonged Neonatal Stay	
Author	≤3 h	>3 h	≤3 h	>3 h	≤3 h	>3 h	≤3 h	>3 h	>3 h	≤3 h	≤3 h	>3 h	≤3 h	>3 h	≤3 h	>3 h	≤3 h	>3 h
2007; Cheng	60/4901	11/257	17/4901	1/257	NR	NR	31/4901	3/257	NR	NR	NR	NR	1061/4901	73/257	145/4901	14/257	445/4901	34/257
2009; Allen	311/64,586	17/968	NR	NR	NR	NR	NR	NR	553/64,586	27/968	NR	NR	NR	NR	2409/64,586	90/968	NR	NR
2017; Ogunyemi	NR	NR	NR	NR	NR	NR	NR	NR	NR	NR	NR	NR	290/5759	12/276	410/5700	40/278	NR	NR
2019; Infante	3/2081	0/64	NR	NR	37/1858	3/54	NR	NR	NR	NR	39/2081	3/64	NR	NR	NR	NR	NR	NR
Egger Bias (*p*-value)	NC												NC		NC			
I^2^ 95% CI	0% (0.0%–72.9%)												NC		0% (0.0%–72.9%)			
Q Cochran (*p*-value)	0.987												0.121		0.417			
OR 95% CI	**3.67 (2.49–5.43)**												**1.29 (1.01–1.66)**		**2.41 (2.02–2.88)**			
	**Minor Trauma**				**Major Trauma**				**Shoulder Dystocia**		**Composite Neonatal Morbidity**				**Any Perinatal Morbidity**			
**Author**	**>3 h**		**>3 h**		**≤3 h**		**>3 h**		**≤3 h**	**>3 h**	**≤3 h**		**>3 h**		**≤3 h**		**>3 h**	
2007; Cheng	NR		NR		NR		NR		117/4901	10/257	361/4901		33/257		NR		NR	
2009; Allen	586/64,586		27/968		104/64,586		2/968		NR	NR	NR		NR		3662/64,586		128/968	
2017; Ogunyemi	NR		NR		NR		NR		NR	NR	NR		NR		NR		NR	
2019; Infante	NR		NR		NR		NR		NR	NR	70/2081		6/64		NR		NR	
Egger Bias (*p*-value)							NC											
I^2^ 95% CI							NC											
Q Cochran (*p*-value)							0.330											
OR 95% CI							**1.97 (1.39–2.80)**											

NR: not reported; NR: not calculated; CI: confidence interval; Random effects (DerSimonian–Laird); Bold: Significant results are highlighted.
